# Altered responsiveness to extracellular ATP enhances acetaminophen hepatotoxicity

**DOI:** 10.1186/1478-811X-11-10

**Published:** 2013-02-05

**Authors:** Sylvia S Amaral, André G Oliveira, Pedro E Marques, Jayane L D Quintão, Daniele A Pires, Rodrigo R Resende, Bruna R Sousa, Juliana G Melgaço, Marcelo A Pinto, Remo C Russo, Ariane K C Gomes, Lidia M Andrade, Rafael F Zanin, Rafaela V S Pereira, Cristina Bonorino, Frederico M Soriani, Cristiano X Lima, Denise C Cara, Mauro M Teixeira, Maria F Leite, Gustavo B Menezes

**Affiliations:** 1 Laboratório de Imunobiofotônica, Departamento de Morfologia, UFMG, Belo Horizonte, MG, Brazil; 2 Departamento de Bioquímica e Imunologia, UFMG, Belo Horizonte, MG, Brazil; 3 Instituto Oswaldo Cruz, Fiocruz, Rio de Janeiro, Brazil; 4 Departamento de Fisiologia e Biofísica, UFMG, Belo Horizonte, MG, Brazil; 5 Instituto de Ciências Biomédicas, Pontifícia Universidade Católica, Porto Alegre, RS, Brazil; 6 Departamento de Biologia Geral, UFMG, Belo Horizonte, MG, Brazil; 7 Departamento de Cirurgia, Faculdade de Medicina, UFMG, Belo Horizonte, MG, Brazil; 8 Howard Hughes Medical Institute, Chevy Chase, MD, USA; 9 ICB-UFMG, Av. Antonio Carlos, 6627 Pampulha, Belo Horizonte, MG, Brazil

**Keywords:** Liver injury, Sterile inflammation, Acetaminophen, Remote injury, Cell death, Immune system, Purinergic signaling, Inflammation

## Abstract

**Background:**

Adenosine triphosphate (ATP) is secreted from hepatocytes under physiological conditions and plays an important role in liver biology through the activation of P2 receptors. Conversely, higher extracellular ATP concentrations, as observed during necrosis, trigger inflammatory responses that contribute to the progression of liver injury. Impaired calcium (Ca^2+^) homeostasis is a hallmark of acetaminophen (APAP)-induced hepatotoxicity, and since ATP induces mobilization of the intracellular Ca^2+^ stocks, we evaluated if the release of ATP during APAP-induced necrosis could directly contribute to hepatocyte death.

**Results:**

APAP overdose resulted in liver necrosis, massive neutrophil infiltration and large non-perfused areas, as well as remote lung inflammation. In the liver, these effects were significantly abrogated after ATP metabolism by apyrase or P2X receptors blockage, but none of the treatments prevented remote lung inflammation, suggesting a confined local contribution of purinergic signaling into liver environment. *In vitro*, APAP administration to primary mouse hepatocytes and also HepG2 cells caused cell death in a dose-dependent manner. Interestingly, exposure of HepG2 cells to APAP elicited significant release of ATP to the supernatant in levels that were high enough to promote direct cytotoxicity to healthy primary hepatocytes or HepG2 cells. In agreement to our *in vivo* results, apyrase treatment or blockage of P2 receptors reduced APAP cytotoxicity. Likewise, ATP exposure caused significant higher intracellular Ca^2+^ signal in APAP-treated primary hepatocytes, which was reproduced in HepG2 cells. Quantitative real time PCR showed that APAP-challenged HepG2 cells expressed higher levels of several purinergic receptors, which may explain the hypersensitivity to extracellular ATP. This phenotype was confirmed in humans analyzing liver biopsies from patients diagnosed with acute hepatic failure.

**Conclusion:**

We suggest that under pathological conditions, ATP may act not only an immune system activator, but also as a paracrine direct cytotoxic DAMP through the dysregulation of Ca^2+^ homeostasis.

## Lay abstract

The abusive use of medications is a major health issue and accounts every year to huge hospitalization costs and several deaths. Analgesics are one of the first alternatives to treat fever and pain, and acetaminophen is the most frequent drug found in these formulations. It is not surprising that the cases of acetaminophen overdose are still frequent in the clinics, and since the liver is a central organ in acetaminophen detoxification, hepatocytes are directly damaged during overdose. Despite the liver ability to regenerate after injury, a massive cell death may trigger an inflammatory response that accounts to additional injury. One of the strategies to restrict organ injury is to control liver inflammation, avoiding organ failure. Here we show that ATP, a key molecule in cell bioenergetics, is also involved in liver inflammation. When cells die, they release ATP to the extracellular environment and this may directly cause additional cell hepatocyte death. These effects may be explained by ATP ability to directly cause intracellular ionic dysregulation in acetaminophen-treated cells. Mice that received an overdose of acetaminophen had significantly less liver damage when extracellular ATP actions were inhibited. Also, human-derived cells cultivated *in vitro* were also protected from these toxic effects when the same blockage strategy was employed. Finally, we established that liver samples from patients suffering from acute hepatitis expressed more receptors to ATP, which suggests that a similar amplifying effect happened during their disease. In this sense, we provided evidence that managing liver response to extracellular ATP released from dead cells may hold future opportunities to avoid liver failure, transplantations and death.

## Background

Drug-induced liver injury (DILI) is an adverse drug reaction that causes acute hepatocyte death. There are several different grades of DILI, which range from an asymptomatic lesion (detectable only by serum transaminases analysis) to severe cases that require liver transplantation [1]. However, 20% to 50% of eligible patients die before a transplant becomes available as a result of hepatic encephalopathy and multiple organ failure [2], indicating that novel therapies aimed to control the progression of liver damage are extremely necessary. The most common cause of DILI is the overdose of acetaminophen (APAP), a popular antipyretic and analgesic drug. Following APAP administration, its reactive metabolite NAPQI (N-acetyl-p-benzoquinone imine) accumulates within hepatocytes, causing cell death mainly by oncotic necrosis [3].

When cells die under such stressing situations, their intracellular contents are spilled to the interstitium and trigger inflammation by directly causing damage to adjacent cells or activating resident cells to release pro-inflammatory mediators. In the latter case, these molecules are called damage-associated molecular patterns (DAMPs) [4]. In general, immune cells express receptors to almost all molecules that originally inhabit the intracellular compartment [5,6], but not all DAMPs are exclusively associated with immune responses. For example, cells can secrete ATP to modulate intracellular functions, including cytosolic calcium (Ca^2+^) concentration and energetic balance [7-9]. However, extracellular ATP concentration significantly increases during necrosis, which in turn activates inflammasome assembling via P2X7 receptor, leading to release of IL-1β [5,10]. The inflammatory response triggered by necrosis-derived ATP was recently described as an important factor to liver injury progression, and activation of P2X7 receptor is required for manifestations of APAP-induced injury [11].

Impaired intracellular Ca^2+^ management is also observed during APAP-induced hepatotoxicity, and it is closely related to the onset of cell death [12]. Moreover, intracellular Ca^2+^ accumulation, particularly into the nucleus, causes DNA fragmentation by endonucleases, accelerating the progression of APAP-dependent cellular necrosis [13], indicating that molecules with ability to increase Ca^2+^ signaling may cause catastrophic consequences to APAP-challenged cells. ATP induces Ca^2+^ mobilization from intracellular stocks [14] and also by opening Ca^2+^ permeable channels in the membrane via P2 receptors [15]. Taking into account the increased extracellular ATP concentration found during necrosis, we hypothesized that excessive interstitial ATP might contribute to liver injury progression not only via immune system stimulation, but also by worsening intracellular Ca^2+^ imbalance observed during APAP administration, acting as a direct cytotoxic DAMP.

## Results

### Acetaminophen-induced liver damage, but not remote lung inflammation, is dependent on extracellular ATP signaling

**Figure 1:**
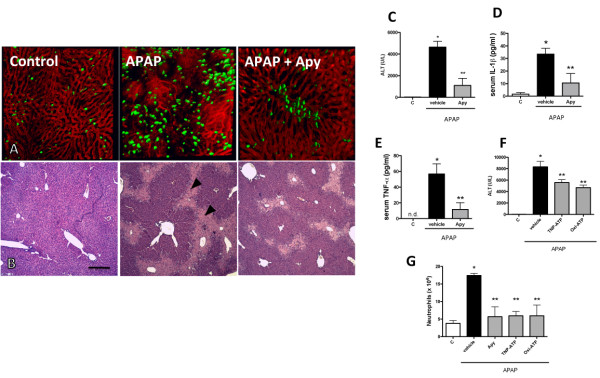
**Extracellular ATP signaling enhances APAP**-**induced hepatotoxicity. **(**A**) Liver intravital microscopy showing sinusoids (in red) and GFP-expressing neutrophils (in green). Mice were treated with acetaminophen (500 mg/Kg; 24 h) and a group received apyrase (25 U/mice) 2 hours after APAP treatment. Control mice were wild type C57 or Lysm-eGFP (in intravital microscopy studies). No significant differences regarding liver injury were observed between C57 and C57-Lysm-eGFP expressing mice. Note the reduced liver injury and neutrophil recruitment in apyrase-treated mice. (**B**) H&E stained liver sections confirmed liver injury and partial protection promoted by extracellular ATP metabolism by apyrase. (**C**) Circulating levels of liver transaminase (ALT) and (**D**-**E**) pro-inflammatory cytokines (TNF-α and IL-1β). (**F**) Serum ALT levels of APAP-challenged mice treated with different purinergic receptors antagonists TNP-ATP (selective P2X, 1 mg/Kg) and oxi-ATP (selective P2X7, 9 mg/Kg). * P < 0.05 in comparison to control group and ** in comparison to vehicle-treated group. N = 5/group. Data are mean ± SEM. Scale: 100 μm.

**Figure 2:**
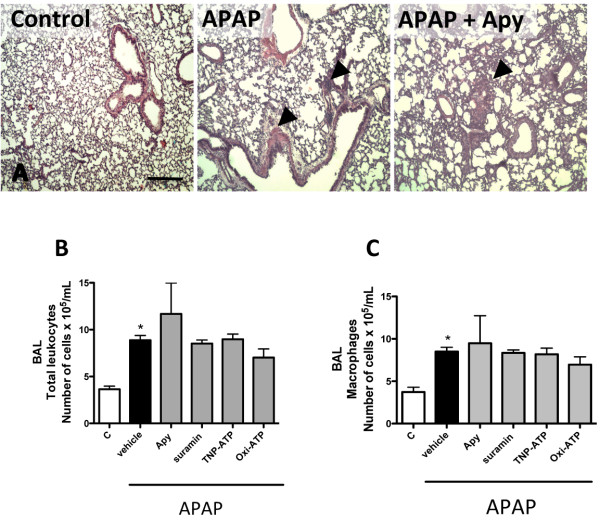
**APAP**-**induced remote lung injury was not reduced following extracellular ATP cleavage or P2 receptors blockage. **(**A**) Lung histology from control mice showing normal morphological findings, while APAP-treated mice presented marked lung inflammation, similarly to apyrase treated group (25 U/mice). Representative images from five different mice/group. (**B**) Leukocyte numbers in bronchial-alveolar lavage (BAL) from APAP treat mice showing that liver injury triggered remote lung inflammation, which was not prevented by dampening ATP sensing. (**C**) Macrophages were the most frequent cell type found in BAL. * - P < 0.05 in comparison to control group, ANOVA followed by Bonferroni post-test. Data are mean ± s.e.m. N = 5/group. Data are mean ± SEM. Scale: 100 μm.

Previous data from our group showed that ATP is released following liver necrosis [16]. Initially, the participation of extracellular ATP in APAP-induced liver injury was investigated by liver confocal intravital microscopy as previously described [16,17]. Control mice presented a fully perfused liver microvasculature, as shown by the regular staining of sinusoids by phycoeritrin (PE)-coupled anti-CD31 (Figure 1A; red channel; Control), and a few neutrophils were found within sinusoids (green channel; Additional file 1: Video 1). However, marked liver necrosis and increased neutrophil infiltration (an indicative of liver inflammation) were observed following 24 hours of APAP administration (500 mg/Kg), revealing also large non-perfused areas (poorly stained by PE-anti-CD31, Figure 1A; APAP; Additional file 2: Video 2). Histopathology analysis (H&E stained slides) confirmed liver necrosis induced by APAP treatment (Figure 1B; control in comparison to APAP; arrow heads), which was diminished following extracellular ATP metabolism by exogenous ATPase (Figure 1B; Apy; apyrase grade VII; 25 U/mice; 24 h). In addition, apyrase significantly reduced liver injury (assessed by serum levels of ALT; Figure 1C) and neutrophil infiltration (Figure 1A; Apy; Additional file 3: Video 3). After APAP administration, increased serum levels of pro-inflammatory cytokines (including TNF-α and IL-1β) were observed, which were significantly reduced by apyrase treatment (Figure 1D-E). Likewise, blockage of P2X (TNP-ATP; 1 mg/Kg; 24 h) or P2X7 (oxi-ATP; 9 mg/Kg; 24 h) (Figure 1F) caused significant reduction in serum levels of ALT and liver injury, which was not reproduced *in vivo* by selective P2Y receptor antagonism (reactive blue-2; 10-100 mg/Kg; 24 h - data not shown). All pharmacological strategies directed to dampen extracellular ATP signaling, including cleavage by apyrase or different P2 receptor antagonists (TNP-ATP and oxi-ATP), reduced liver inflammation, necrosis (as assessed by histological score from H&E slides; Additional file 4: Figure S1), and neutrophil infiltration (Figure 1G). Acute liver injury led to remote lung inflammation (Figure 2A; control in comparison to APAP), with concomitant pulmonary leukocyte accumulation (Figure 2B). Leukocytes recovered from BAL were predominately macrophages (Figure 2C). While dampening ATP sensing resulted in significantly less liver damage and inflammation, no detectable effects on pulmonary injury (Figure 2A) or lung leukocyte infiltration (Figure 2B-C) were observed.

### HepG2 cells release ATP following acetaminophen incubation

**Figure 3:**
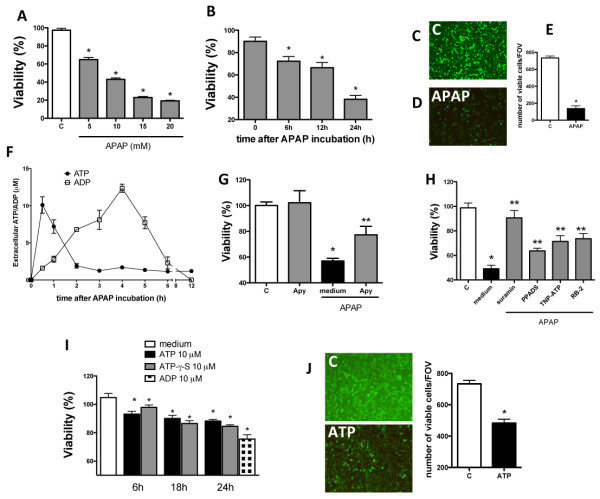
**ATP is released and accounts to additional cell death during APAP incubation. **(**A**-**B**) Dose and time-response curves of APAP challenged HepG2 cells (24 h). (**C**-**E**) Acridine orange/ethidium bromide viability test confirmed APAP cytotoxicity. (**F**) ATP and ADP quantification in HepG2 supernatant (HPLC) in different timepoints after APAP incubation (20 mM). (**G**-**H**) Effects of apyrase (10 U/ml) or different P2, P2X and P2Y blockers (0.1 mM, 100 μM and 30 μM, respectively) on APAP-mediated cell death. (**I**-**J**) Direct cytotoxic effect of ATP and ADP on “naïve” HepG2 cells, confirmed by acridine orange/ethidium bromide viability test. Controls (medium alone) were not different throughout the experiments. * P < 0.05 in comparison to control group and ** in comparison to vehicle or medium treated group.

To expand our *in vivo* findings, we established an *in vitro* model of APAP cytotoxicity using both primary mouse hepatocytes and a human lineage of hepatocytic cells (HepG2 cells). APAP incubation caused cell death (Figure 3A; 5-20 mM; 24 h), which started in the 6^th^ hour post-incubation, reaching 50% of cytotoxicity following 24 hours (Figure 3B; assessed by MTT metabolism). Co-incubation with the standard APAP antidote *N*-acetyl cysteine significantly prevented APAP effects on HepG2 cells, confirming that the major cause of cytotoxicity in our *in vitro* model was due to APAP bioactivation to toxic metabolites (data not shown). APAP-mediated cytotoxicity was also evaluated by an alternative cell viability test (ethidium bromide/acridine orange staining). Incubation with APAP (20 mM; 24 h) caused significant reduction in the number of viable cells in comparison to controls (Figure 3C-E). Therefore, these settings were chosen to all subsequent *in vitro* experiments.

To prove that stressed/necrotic hepatocytes could be a relevant source of extracellular ATP, we incubated HepG2 cells with APAP and measured ATP and ADP concentrations in the supernatant by high-performance liquid chromatography (HPLC). Supernatant from untreated cells had undetectable amounts of both ATP and ADP. However, following APAP incubation, extracellular ATP concentration rapidly increased, while ADP was detected in later timepoints (Figure 3F). Interestingly, cleavage of extracellular ATP reverted APAP cytotoxicity (Figure 3G), which was reproduced by unspecific blockage of P2 receptors by suramin (0.01-0.1 mM; 24 h) (Figure 3H). Selective participation of different P2 receptors subfamilies was also investigated. Specific P2X receptors antagonism by PPADS (0.1-100 μM; 24 h) or TNP-ATP (0.1-100 μM) also partially prevented APAP cytotoxicity, as well as selective blockage of P2Y receptors by reactive blue-2 (3-30 μM) (Figure 3H). While selective P2X7 blockage (by oxi-ATP) prevented APAP hepatotoxicity *in vivo*, no detectable effects were observed *in vitro* using a large dose range (10-100 μM; 24 h) (Additional file 5: Figure S2). These data suggest that not a specific subtype, but several ATP and ADP receptors may be involved in extracellular purinergic signaling during necrosis, and their combined stimulation may enhance cell death independently of the immune system activation.

In this context, we hypothesized that ATP might be directly harmful in concentrations that are biologically relevant. In fact, in the same titers found in medium recovered from APAP-challenged cells, both ATP and ADP were directly cytotoxic to “naïve” HepG2 cells (10 μM, Figure 3I, Additional file 4: Figure S1). ATP-mediated toxicity was detected in early timepoints (6 h), persisting until the end of incubation period (24 h, Figure 3I), which was also confirmed by ethidium bromide/acridine orange staining (Figure 3J; control in comparison to APAP). Subsequently, we examined whether the effects observed following ATP incubation were derived only from its metabolism to ADP. For this, we used a non-hydrolysable ATP (ATP-γ-S) in the same dose range and confirmed that this stable analogue was equally able to deflagrate cell death (Figure 3I).

### Increased intracellular Ca^2+^ availability underlies the mechanisms by which ATP/P2 receptors activation contribute to APAP-mediated cell death

**Figure 4:**
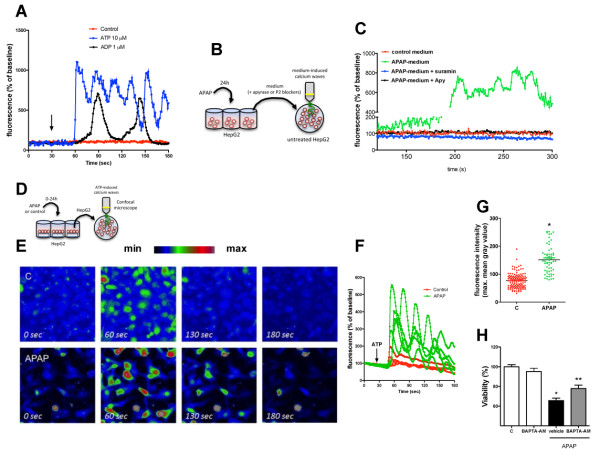
**Hyper**-**responsiveness to ATP increased intracellular Ca**^**2 **+^**signal during APAP challenge. **(**A**) Ca^2+^ signal induced by ATP and ADP (10 μM) in HepG2 cells. (**B**-**C**) Culture medium from necrotic/suffering cells triggered intracellular Ca^2+^ signal in “naïve” HepG2 cells, which was abolished by apyrase (10 U/ml) or P2 blocker suramin (0.1 mM). (**D**-**E**) Snapshots from confocal microscopy showing Ca^2+ ^signal in HepG2 cells treated or not with APAP at 20 mM. (**F**-**G**) ATP-triggered Ca^2+^ signals from cells incubated or not with APAP for 24 hours. (**H**) Ca^2+^ sequestration by BAPTA-AM (1 nM) partially reverted APAP-mediated cell death. * P < 0.05 in comparison to control group and ** in comparison to vehicle or medium treated group.

To investigate the biological relevance of extracellular purines during necrosis, we measured the Ca^2+^ signal amplitude by confocal microscopy when HepG2 cells were challenged with ATP or ADP in the same concentration range found during APAP-mediated cytotoxicity (10 μM). Following ATP or ADP administration, marked intracellular Ca^2+^ signals were observed in comparison to controls, which were sustained and repeated throughout the incubation time (Figure 4A). Strikingly, “naïve” HepG2 cells that received supernatant collected from APAP-treated cells (Figure 4B) also displayed enhanced intracellular Ca^2+^ signal, which was completely abolished by incubation with apyrase or unspecific P2 receptor antagonist suramin (Figure 4C). These data suggest that such extracellular concentrations of ATP and ADP may play an important role in intracellular Ca^2+^ mobilization in during APAP incubation.

Next, we investigated if acetaminophen challenge was also able to modify cell responsiveness to extracellular ATP released during necrosis. For this, we cultured HepG2 cells in the presence or absence of APAP (20mM) for 24 hours (Figure 4D). Original medium was removed and intracellular Ca^2+^ signal was triggered by the same extracellular ATP concentration found during APAP treatment (10 μM). Following ATP administration, the remaining viable APAP-treated cells displayed higher, sustained and repeated Ca^2+^ signals (Figure 4E-F, Additional file 6: Video 4), while control cells had lower intracellular Ca^2+^ increase and rapidly returned to baseline values (Figure 4E-F, Additional file 7: Video 5). Additionally, we observed that not only APAP-treated cells were hyper-responsive to ATP, but the majority of the reactive cells reached higher fluorescence values than untreated cells, indicating a both quantitative and qualitative change in Ca^2+^ dynamics during APAP incubation (Figure 4G).

To confirm that unbalanced calcium signal was involved in APAP cytotoxicity, we treated HepG2 cells with an intracellular Ca^2+^ scavenger (BAPTA-AM; 1 nM) throughout APAP challenge. Incubation with BAPTA-AM reduced APAP-mediated cell death in 50% (Figure 4H), suggesting that increased intracellular calcium availability contributed to APAP-mediated cell death.

### Primary mouse hepatocytes also developed hyper-responsiveness to extracellular ATP following APAP exposure, which is prevented by P2 receptor antagonism

**Figure 5:**
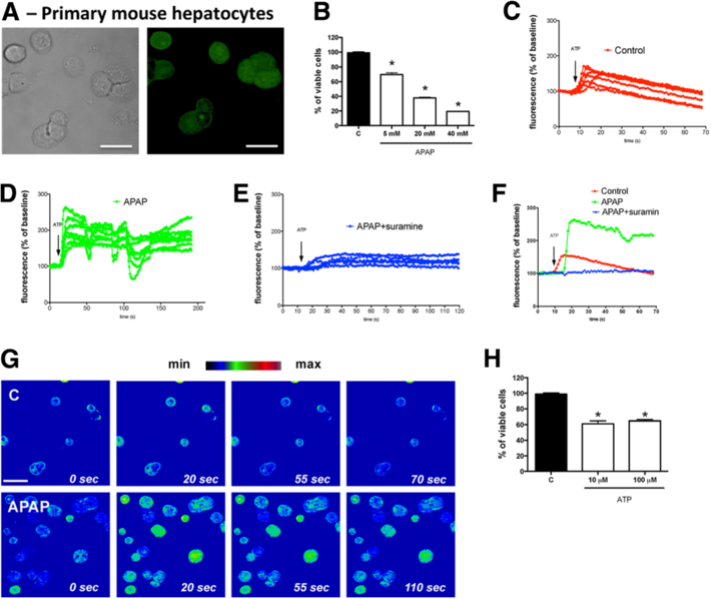
**APAP**-**challenged primary hepatocytes presented sustained and repeated intracellular calcium signal due to exogenous ATP administration. **(**A**) Primary mouse hepatocytes (PMH) were isolated and loaded with a fluorescent calcium probe (Fluo4-AM). (**B**) Following APAP incubation (18 h), PMH viability decreased in a dose-dependent manner, reaching 50% of survival when 20 mM of APAP was used. Therefore, this dose was used to subsequent experiments. (**C**) ATP administration (10 μM) caused calcium signal in naïve hepatocytes, which returned to baseline values after 60 seconds. Six replicates are represented in the graph. (**D**) However, APAP-treated cells (20 mM; 6 h) developed a hyper-responsive behavior to the same ATP dose, displaying higher and sustained calcium signal, which was also prolonged for longer periods (200 seconds). (**E**) Treatment with an unspecific P2 antagonist (suramin, 0.1 mM) completely abrogated APAP effects over ATP stimulation. (**F**) Representative cells of each group were displayed together. (**G**) Snapshots from live calcium signal recording using confocal microscopy. Videos were rendered in “rainbow pallet” to facilitate fluorescence observation (generated by Fluo4-AM). Following 110 seconds of ATP stimulation, APAP treated cells remaining responsive with increased intracellular calcium signal, while control cells returned to baseline values after 55–70 seconds. Scale = 20 μm. (**H**) Incubation of primary hepatocytes with exogenous ATP in the dose range found during necrosis (10-100 μm, 18 h) significantly reduced cell viability. * P < 0.05 in comparison to control group. Data are mean ± SEM.

To validate our data obtained in HepG2 cells, we obtained primary hepatocytes from mice (Figure 5A) and incubated with different APAP and ATP doses (5-40 mM and 10-100 μM, respectively). As shown in Figure 5B, APAP incubation decreased cell viability (18 h) in a dose dependent manner. Incubation with APAP (20 mM) caused 50% of cell death, and this dose was chosen for subsequent experiments. Following exogenous ATP administration in the same dose found during necrosis (10 μM), naïve mouse hepatocytes incubated with APAP (20 mM; 6 h) increased intracellular calcium signal, and returned to baseline values after 60 seconds (Figure 5C and 5G; Additional file 8: Video 6). However, APAP-treated primary hepatocytes presented prolonged (Additional file 9: Video 7) or repeated intracellular calcium signal (Figure 5D and 5G; Additional file 10: Video 8), reaching higher fluorescence values in both cases. APAP-treated primary hepatocytes remained responsive to exogenous ATP for longer periods (~200 seconds; 3-fold increase; Figure 5D) in comparison to controls (~70 seconds, Figure 5C). ATP-triggered calcium increase was totally abrogated when APAP-challenged cells were treated with a P2R blocker (suramin; Figure 5E; Additional file 11: Video 9), suggesting that, analogous to HepG2 cells, APAP incubation also caused hyper-responsiveness to extracellular ATP in primary mouse hepatocytes. Moreover, incubation of primary hepatocytes with exogenous ATP in the dose range found during necrosis (10-100 μm, 18 h) significantly reduced cell viability, confirming that in biological relevant concentrations extracellular ATP may be also directly cytotoxic to mouse liver cells (Figure 5H).

### Increased expression of several purinergic receptors may explain the hyper-responsiveness to ATP during necrosis

**Figure 6:**
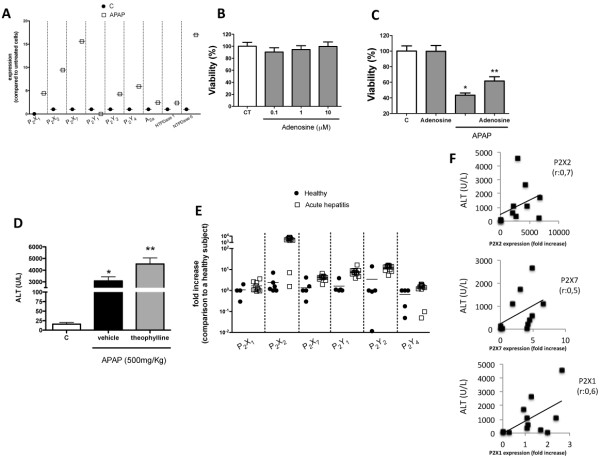
**Up**-**regulation of several purinergic receptors may explain hyper**-**responsiveness to ATP during hepatotoxicity. **(**A**) Quantitative PCR for purinergic receptors from cells cultivated in the presence or absence of APAP (20 nM; 24 h). Untreated HepG2 cells (C) determined baseline expression. (**B**-**C**) HepG2 incubation with adenosine in the presence or absence of APAP. (**D**) Serum ALT levels of APAP-challenged mice treated with theophylline (P1 receptor blocker, 20 mg/Kg). (**E**) Quantitative PCR analysis of liver samples from acute hepatitis patients in comparison to healthy volunteers. Baseline expression was determined by choosing a healthy volunteer as control. (**F**) Changes in expression of different P2 receptors were compared to serum ALT levels from healthy and acute hepatitis patients. Note that increased P2R expression was correlated with higher serum ALT levels in several patients. Pearson’s correlation was calculated comparing fold increase of P2R expression to serum ALT levels (r). * P < 0.05 in comparison to control group and ** in comparison to vehicle treated group.

In order to elucidate the mechanisms involved in the increased sensitivity to extracellular purines displayed by APAP-treated cells, we measured the expression of several purinergic receptors. Quantitative PCR analysis revealed that numerous P2 receptors (P2X1, P2X2, P2X7, P2Y2, P2Y4) and ectonucleotidases (NTPDase 1 and 6) were upregulated during APAP incubation in comparison to controls (Figure 6A), which could be one of the reasons for the elevated Ca^2+^ signal triggered by ATP in APAP-incubated cells. Taking into account the detrimental effects of an exacerbated extracellular ATP signaling during necrosis, we hypothesized that the elevated expression of NTPDases might consist in a mechanism for ATP signaling restriction and cell protection, driving extracellular ATP metabolism ultimately to adenosine. In fact, *in vitro* incubation with adenosine was not only innocuous to HepG2 cells (Figure 6B), but also partially reverted APAP-mediated cytotoxicity (Figure 6C). Corroborating this protective loop, an increased expression of adenosine A2a receptor (A2aR) was observed in APAP-challenged cells. In fact, A2aR is described to reduce inflammatory reactions and accelerate healing when binding to adenosine [18]. Such protective profile was also confirmed *in vivo*, since blockage of adenosine receptors with an unspecific P1 antagonist (theophylline) worsened APAP-mediated liver injury (Figure 6D).

To validate our findings in humans, we investigated the profile of purinergic receptors expression in patients diagnosed with drug-induced acute hepatitis. In agreement, several P2 receptors were also upregulated in acute hepatitis patients in comparison to healthy donors (Figure 6E), suggesting that altered cell responsiveness to extracellular purines may be also relevant in the context of liver injury progression. In addition, enhanced purinergic receptor expression (P2X2, P2X7 and P2X1; Figure 6F) was correlated with higher grades of liver injury (assessed by serum ALT levels) in several acute hepatitis patients (Pearson’s correlation; r).

## Discussion

Extracellular ATP is a well-characterized damage-associated molecular pattern (DAMP), which activates the NLRP3 inflammasome via P2X7 receptors, inducing production of inflammatory cytokines, including interleukin-1β [5,11,19,20]. The majority of *in vitro* studies commonly use elevated concentrations of ATP (ranging from 1 to 10 mM) and short incubation regimes to induce cell activation, particularly leukocytes [21-23]. However, these conditions are unlikely to represent any *in vivo* environment found under either physiological or pathological circumstances [24]. For instance, extracellular ATP concentration increases during inflammatory responses, but it reaches maximum concentrations in the range of hundred micromolars [25]. So far, the damage exerted by increased extracellular concentrations of ATP has been solely attributed to indirect, inflammatory effects over immune cells [11,26]. In this study, we showed that ATP and ADP have direct cytotoxic effects in hepatic cells and may have profound influence in the pathogenesis of acute liver failure. We provided evidence that ATP signaling during necrosis might have a double-faceted action by i) enhancing inflammatory response via IL-1β release and ii) directly causing hepatotoxicity due to a hyper-responsiveness behavior to ATP and increased intracellular Ca^2+^ availability.

In this study, we used a murine model of APAP poisoning to show that activation of several P2 receptors is detrimental during liver injury progression. Confocal intravital microscopy revealed remarkable changes in the liver environment during APAP overdose that were dependent on extracellular ATP signaling. Also, harmful effects of P2 receptor agonists seems to be restricted to the liver, since blockage of ATP/ADP sensing was not effective in preventing remote lung injury. In fact, despite the hepatoprotection and the reduced levels of circulating cytokines promoted by apyrase treatment, DAMPs released probably from remote injury were sufficient to support lung inflammation [17]. In line with this interpretation, recent work demonstrated that increased serum levels of ATP found during hepatectomy were only transient (~ 5 minutes) and rapidly returned to baseline values [27]. These data indicate that while extracellular ATP and ADP play a key role in local liver injury, they are not mediators of remote inflammatory responses.

Also, we performed a series of *in vitro* experiments to determine if hepatocytes could constitute a relevant source of extracellular ATP. Subsequently to APAP incubation, higher concentrations of ATP and ADP were found in the culture medium recovered from stressed/necrotic HepG2 cells in comparison to controls. Taking into account that these titers were sufficient to directly induce HepG2 death, we postulated that ATP released from a suffering or necrotic cell binds to P2 receptors in a challenged, neighboring cell and increases its intracellular Ca^2+^ concentration in sufficiently high levels to accelerate cell damage or even death. It is possible that off-target factors, including alterations in probe hydrolysis or intracellular pH variations could contribute to Fluo-4AM fluorescence variations observed in the current study. However, these factors possibly had a minor impact in our results, as we were able to significantly abrogate ATP-mediated increase in intracellular calcium signal with P2R antagonist treatment. Therefore, our data suggest that the main pathway involved in altered cell responsiveness in our model is related to P2R signaling. In fact, paracrine communication between hepatocytes mediated by ATP was previously reported [28], and blockage of cell-cell interaction via gap junctions may hold opportunities to restrict liver damage during APAP overdose [29]. Also, recent data have confirmed that extracellular ATP may enhance APAP-mediated liver damage *in vivo* via activation of P2X7 and P2Y2 [30,31]. Thus, we suggest that necrotic/suffering hepatocytes may efficiently supply extracellular ATP to fuel both immune system activation and direct hepatotoxicity. Likewise, *in vitro* effects were not restricted to single receptor activation (e.g. P2X7), but rather mediated by different P2 receptors subfamilies, suggesting that larger spectrum inhibition might be necessary to promote appreciable cytoprotection.

ATP induces intracellular signals by mobilizing Ca^2+^ from cytosolic and nuclear stocks [14] or allowing its influx across the membrane [32]. Interestingly, in situations of deprivation of mitochondrial ATP, as seen throughout APAP challenge, cells may upregulate P2 receptors to increase intracellular Ca^2+^ and stimulate ATP synthesis within mitochondria as a tentative to escape from an irreversible damage [7-9]. Accordingly, both primary mouse hepatocytes and HepG2 cells developed a hyper-responsive behavior to exogenous ATP following APAP administration, which may be correlated with increased expression of different purinergic receptors. Therefore, we propose that such change in P2R levels observed in both APAP-treated cells and in liver biopsies from acute hepatitis patients may be a strategy to restrict cell suffering through P2 receptors-mediated intracellular Ca^2+^ increase. However, APAP challenge may disturb important intracellular mechanisms that manage Ca^2+^ compartmentalization, since challenged cells displayed elevated intracellular Ca^2+^ signal in our *in vitro* model. In this context, cytoprotection promoted by intracellular calcium sequestration (using BAPTA-AM) provided interesting insights for future investigations focused on elucidate the most important calcium sources that extracellular ATP recruits to boost APAP-triggered hepatotoxicity. It is worth taking into consideration that although we observed P2 receptor upregulation in HepG2 cells following APAP challenge, it is conceivable that purinergic signaling in other cell types (i.e. liver resident cells and infiltrating leukocytes) is also involved in the development of acute liver failure caused by APAP administration. Furthermore, although we confirmed APAP cytotoxicity by two different methodologies, alterations in cell metabolic pathways following APAP overdose (i.e. mitochondrial activity and cell proliferation rate) may also contribute to the mechanisms involved in APAP cytotoxicity.

## Conclusions

**Figure 7:**
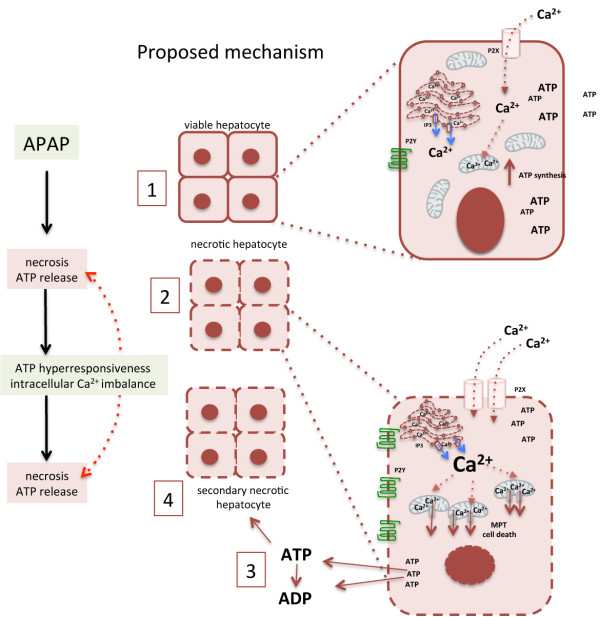
**Proposed mechanism: 1: Under physiological conditions, extracellular ATP regulates several intracellular signaling pathways, which involves also calcium compartmentalization.** 2–3: Acetaminophen incubation directly causes hepatocyte necrosis, calcium imbalance and further ATP release. 4: In parallel, challenged viable hepatocytes up-regulate several purinergic receptors, probably as a regulatory homeostatic strategy, causing ATP hyper-responsiveness. Binding of extracellular ATP to purinergic receptors increases intracellular Ca^2+ ^and pulses, which accounted to additional cell necrosis, reverberating APAP-induced death. Dampening of extracellular ATP signalling or reducing intracellular Ca^2+ ^availability significantly reduced hepatocyte necrosis. Data from FHF patients suggest that a similar necrosis-amplification pathway may be involved in organ injury progression.

We provided novel evidence that within the liver environment ATP acts not only as an immune system activator, but also as a direct cytotoxic DAMP by increasing intracellular Ca^2+^ concentration. Also, while upregulation of P2 receptors may consist in a physiological strategy to regulate cell functions, the hyper-responsiveness behavior to biologically relevant concentrations of ATP accounted to additional hepatotoxicity (Figure 7). These findings have clear implications for liver disease pathogenesis and therapy, providing rationale to alternative pharmacological approaches for management of acute hepatitis.

## Methods

### Mice

C57BL/6 mice were from Centro de Bioterismo in UFMG (Brazil). Lysm-eGFP mice were donated by Dr. Paul Kubes (University of Calgary, Canada). All procedures were approved by Animal Care and Use Committee in UFMG (CEBIO n°051/2011). The investigation conformed to the standards of *Guide for the Care and Use of Laboratory Animals* (National Institutes of Health publication 85–23, 1996 revision).

### Model of APAP-induced liver injury

Mice were fasted for 15 hours before oral APAP administration (500 mg/kg; Sigma, USA) or vehicle (warm sterile saline). After different time-points, mice were anesthetized and euthanized for blood (serum), liver, BAL (bronchial-alveolar lavage) and lung harvesting. ALT determination was performed using a kinetic test (Bioclin, Brazil) and cytokines and chemokines were quantified by ELISA kits (R&D systems, USA) both in serum and tissues. Fragments of liver and lung were fixed in formalin and sectioned for histology (H&E). Histological score was assessed by an experienced pathologist, in which 0: no lesion present; 1/2: individual necrotic cells seen at the first cell layer adjacent to the central vein, and hyaline degeneration present; 1: necrotic cells extending two or three cell layers from the central veins; 2: necrotic cells extending three to six cell layers from the central veins, but limited in peripheral distribution; 3: the same as 2, but with necrosis extending from one central vein to another; 4: more severe than 3, with extensive centrilobular necrosis throughout the section. A final score was given for each liver section [33]. Serum mitDNA (cytochrome C primer) was estimated by Real-Time PCR as previously described [34]. Neutrophil infiltration into the liver was estimated by the myeloperoxidase activity assay (MPO) [35]. In a separated set of experiments, the liver was imaged using confocal intravital microscopy as described previously [16]. Three-dimensional video reconstructions were made using confocal Z-stacks (40 μm; 1 μm step) and mounted using Volocity software (Perkin-Elmer, USA). BAL was collected for leukocyte counting [36]. All experimental groups included N ≥ 5.

### In vivo drugs and treatments

Mice received APAP (500 mg/Kg; Sigma) by oral gavage diluted in warm saline. This dose of APAP is almost completely metabolized within 1.5-2 hours [37]. Therefore, all pharmacological treatments were performed two hours after APAP gavage to avoid interference in APAP bioactivation and off-target effects. Suramin (5 mg/Kg; i.v.), TNP-ATP (1 mg/Kg; i.v.), oxidized-ATP (oxi-ATP; 9 mg/Kg; i.v.), reactive blue-2 (10-100 mg/Kg; i.p.), apyrase (25 U/mice; i.v.) and theophylline (20 mg/Kg) were dissolved in sterile saline following supplier instructions (Sigma, USA).

### In vitro HepG2 assays

HepG2 (American Type Culture Collection) cells were maintained at 37°C under an atmosphere of 5% CO2 in complete RPMI1640 medium containing 10% FBS and cultured in 10^5^ cells/well in 96 wells plates. After 24 hours of incubation the supernatant was replaced by medium without FBS containing the treatments [38]. APAP (5-20 mM), suramin (0.01-0.1 mM), TNP-ATP (0.1-100 μM), PPADS (0.1-100 μM), oxi-ATP (10-1000 μM), apyrase grade IV (10 U/mL), reactive blue-2 (3-30 μM, a generous gift from Dr. Tomoyuki Saino, Iwate Medical University, Japan), ATP (1-100 μM), ATP-γ-S (10 μM), ADP (1-10 μM), adenosine (0.1-10 μM) and BAPTA-AM (1 nM) were dissolved in DMSO or water following supplier instructions (Sigma, USA), and added into culture medium to incubation throughout the experiments. Working doses were chosen based on dose-response curves previously established in our group (Additional file 5: Figure S2). Cell viability was assessed by MTT metabolism assay (Sigma, USA) or by ethidium bromide/acridine orange viability assay [39]. Purinergic receptors and ectonucleotidases expression was quantified by Real-Time PCR as described previously [40,41]. Experiments were repeated at least three times, using 12 replicates per group.

### Primary mouse hepatocytes (PMH) isolation and culture

PMH were isolated as previously described [42,43]. Briefly, the portal vein was cannulated, and a solution collagenase B (4 mg/ml; Roche; from *C*. *histolyticum*) was perfused during 8 minutes (5 ml/min). Following initial digestion, liver was separated from the diaphragm and stomach, and carefully minced in a plastic Petri dish in Williams’s medium and the resultant cells were double filtered in serial nylon mesh filters. The cell suspension was washed twice with William’s medium and ressuspended in William’s medium containing 50 U of penicillin and 50 mg of streptomycin. This protocol yielded a high rate of viable hepatocytes (>95% as assessed by commercial fast hematological staining kit and Trypan blue exclusion). Cells were then seeded on collagen I-treated glass coverslips (3 × 10^5^ cells/well) and cultured using 6-wells plates to further MTT and calcium signal assays (as described previously for HepG2 cells).

### Analysis of extracellular nucleotides by HPLC

Cells were incubated in presence or absence of APAP and supernant was recovered in different timepoints. Aliquots of 40 μL were applied to a reverse phase HPLC system using a C18 Shimadzu column (Shimadzu, Japan) with absorbance measured at 260 nm. The mobile phase was 60 mM KH_2_PO_4_, 5 mM tetrabutylammonium chloride, pH 5.9, in 15% methanol. Retention times were assessed using standard samples of ATP and its metabolites.

### Detection of intracellular Ca^2+^ signals with confocal microscopy

Nuclear and cytosolic Ca^2+^ were monitored in individual cells by using time-lapse confocal microscopy, as described [14]. Cells were incubated with 4 μM cell permeant fluo-4-AM (fluo-4 acetoxymethyl ester; Molecular Probes) and all fluorescence analyses were performed offline using ImageJ software (NIH) as previously described [14].

### Human patients

Fifteen patients diagnosed with non-viral and suspected of drug-induced acute liver failure addressed to the Liver Clinic of Hospital Federal de Bonsucesso of Rio de Janeiro were enrolled for this study (Table 1). Inclusion criteria demanded the development of coagulopathy (prothrombin time activity > 15 s) or international normalized ratio (INR) ≥ 1.5, and hepatic encephalopathy within 8 weeks of jaundice onset in the absence of pre-existing liver disease. Liver samples were also collected from 5 healthy subjects (liver donors) for the experiments. Expression of purinergic receptors and ectonucleotidases were quantified by Real-Time PCR as described previously [40,41]. Liver samples were collected and immediately frozen in liquid nitrogen and kept in –80°C until processing for PCR analysis. This study was approved by Institutional Review Boards (CEP-Fiocruz 22/03) and performed under an informed consent by all participants. The study protocol conformed to the ethical guidelines of the 1975 Declaration of Helsinki.

**Table 1:** Clinical data of patients enrolled in the study

**Patient ID**	**Age**	**ALT (U/L)**	**INR**	**serum bilirubin (mg/dl)**
*Acute Hepatitis *(*n* = *15*)	6-55	122-7300	3.3-18.2	3.5-37
*Healthy donors *(*n* = *5*)	25-38	<40	ND	ND

Serum levels of liver transaminase (ALT) and bilirubin from patients enrolled in the study. Liver biopsies were collected during transplantation procedures. INR: International Normalized Ratio, ND: non-determined.

### Statistics

Statistical analyses were performed using one-way ANOVA (Dunnett or Bonferroni post-test) or Student’s *t* test. *P* values less than 0.05 were considered statistically significant. Pearson’s correlation was calculated by Excel 2011 for Mac (Microsoft). All data are presented as mean ± SEM.

## Abbreviations

ATP: Adenosine triphosphate; APAP: Acetaminophen; DAMPs: Damage-associated molecular patterns; mitDNA: Mitochondrial DNA; BAL: Broncho-alveolar lavage; ALT: Alanine aminotransferase; MPO: Myeloperoxidase; i.v.: Intravenous; H&E: Hematoxilin and eosin; MTT: Thiazolyl blue tetrazolium bromide; HPLC: High performance liquid chromatography; Lysm-eGFP: Lysozyme M promoter for enhanced green fluorescent protein; PECAM-1: Platelet-endothelial cell adhesion molecule-1; Q-PCR: Quantitative polymerase chain reaction.

## Competing interests

The authors declare that they have no competing interests.

## Authors’ contributions

SSA, AGO, PEM, JLDQ, DAP, RVSP and LMA performed *in vivo* and *in vitro* experiments. BRS, RRR, RFZ, CB and FMS performed PCR and HLPC experiments. CXL, JGM and MAP collected and processed human samples and clinical data. RCR and AKCG performed lung injury assays. GBM, MMT, MFL and DCC designed the experiments and discussed data. GBM and MFL wrote the paper. All authors have read and approved the final manuscript.

## Supplementary Material

Additional file 1: Video 1 Three-dimensional *Z-stack *rendering from liver confocal intravital microscopy – Control mouse. Lysm-eGFP mice were used to visualize neutrophil infiltration and sinusoidal perfusion. Sinusoids were stained by i.v. injection of PE-coupled anti-PECAM-1. *Z*-stacks were made (depth: 40 μm) and TIFF-acquired images were mounted by using Volocity software (NIH, USA). Mice were imaged by sequential laser scans (2.71 seconds). Click here for file

Additional file 2: Video 2 Three-dimensional *Z-stack *rendering from liver confocal intravital microscopy – Acetaminophen-treated mouse. Lysm-eGFP mice were treated with acetaminophen (APAP; 500 mg/Kg; 24 h) and prepared to visualization of neutrophil infiltration and liver sinusoidal perfusion. Sinusoids were stained by i.v. injection of PE-coupled anti-PECAM-1. *Z*-stacks were made (depth: 40 μm) and TIFF-acquired images were mounted by using Volocity software (NIH, USA). Mice were imaged by sequential laser scans (2.71 seconds). Click here for file

Additional file 3: Video 3 Three-dimensional *Z-stack *rendering from liver confocal intravital microscopy – Apyrase treated acetaminophen-challenged mouse. Lysm-eGFP mice were treated with acetaminophen (APAP; 500 mg/Kg; 24 h) and apyrase (25 U/mice, 2 hours after APAP) and prepared to visualization of neutrophil infiltration and liver sinusoidal perfusion. Sinusoids were stained by i.v. injection of PE-coupled anti-PECAM-1. *Z*-stacks were made (depth: 40 μm) and TIFF-acquired images were mounted by using Volocity software (NIH, USA). Mice were imaged by sequential laser scans (2.71 seconds). Click here for file

Additional file 4: Figure S1 H&E slides and histological score from mice treated with different P2R antagonists and challenged with APAP. Mice were treated (2 hours after APAP challenge; 500 mg/Kg; 24 h) with apyrase (25 U/mice), TNP-ATP (1 mg/Kg; i.v.), oxidized-ATP (oxi-ATP; 9 mg/Kg; i.v.) or reactive blue-2 (10-100 mg/Kg; i.p.). Histological score was assessed using 0: no lesion present; 1/2: individual necrotic cells seen at the first cell layer adjacent to the central vein, and hyaline degeneration present; 1: necrotic cells extending two or three cell layers from the central veins; 2: necrotic cells extending three to six cell layers from the central veins, but limited in peripheral distribution; 3: the same as 2, but with necrosis extending from one central vein to another; 4: more severe than 3, with extensive centrilobular necrosis throughout the section. A final score was given for each liver section. * - P < 0.05 in comparison to control (C) group and ** in comparison to vehicle treated group. Data are mean ± SEM. Scale: 100 μm. Click here for file

Additional file 5: Figure S2 Dose–response curves of ATP, ADP and different purinergic receptors antagonists. (A) Dose–response curve ATP and (B) ADP incubation upon HepG2 cells. (C-D) Dose–response curves of suramin (non-selective P2 antagonist; alone and with APAP), (E-F) TNP-ATP (P2X blocker; alone and with APAP), (G-H) PPADS (P2X blocker; alone and with APAP), (I-J) oxi-ATP (specific P2X7 blocker; alone and with APAP) or (K-L) reactive blue-2 (P2Y blocker; alone and with APAP). * - P < 0.05 in comparison to control (C) group and ** in comparison to medium treated group. Click here for file

Additional file 6: Video 4 Detection of intracellular Ca^2+ ^signals with confocal microscopy - APAP treated HepG2 cells. Nuclear and cytosolic Ca^2+ ^were monitored in individual cells by using time-lapse confocal microscopy. HepG2 cells were cultured on glass coverslips in a density of 3×10^5 ^cells/well in 6 wells plates and kept in a Hepes-buffered solution during experiments. Cells were incubated with APAP (10 mM) and after 24 hours 4 μM cell permeant Fluo4-AM (fluo-4 acetoxymethyl ester; Molecular Probes) was added to the culture. ATP (10 μm) was used to trigger intracellular calcium signal. Click here for file

Additional file 7: Video 5 Detection of intracellular Ca^2+ ^signals with confocal microscopy - Control HepG2 cells. Nuclear and cytosolic Ca^2+ ^were monitored in individual cells by using time-lapse confocal microscopy. HepG2 cells were cultured on glass coverslips in a density of 3×10^5 ^cells/well in 6 wells plates and kept in a Hepes-buffered solution during experiments. Cells were incubated with 4 μM cell permeant Fluo4-AM (fluo-4 acetoxymethyl ester; Molecular Probes) was added to the culture. ATP (10 μm) was used to trigger intracellular calcium signal. Click here for file

Additional file 8: Video 6 Detection of intracellular Ca^2+ ^signals with confocal microscopy – Control primary hepatocytes. Nuclear and cytosolic Ca^2+ ^were monitored in individual cells by using time-lapse confocal microscopy. Primary mouse hepatocytes were cultured on glass coverslips in a density of 3×10^5 ^cells/well in 6 wells plates and kept in a Hepes-buffered solution during experiments. Cells were incubated with APAP (20 mM) and after 6 hours 4 μM cell permeant Fluo4-AM (fluo-4 acetoxymethyl ester; Molecular Probes) was added to the culture. ATP (10 μm) was used to trigger intracellular calcium signal. Click here for file

Additional file 9: Video 7 Detection of intracellular Ca^2+ ^signals with confocal microscopy – APAP-treated primary hepatocytes displayed sustained calcium signal following ATP administration. Nuclear and cytosolic Ca^2+ ^were monitored in individual cells by using time-lapse confocal microscopy. Primary mouse hepatocytes were cultured on glass coverslips in a density of 3×10^5 ^cells/well in 6 wells plates and kept in a Hepes-buffered solution during experiments. Cells were incubated with APAP (20 mM) and after 6 hours 4 μM cell permeant Fluo4-AM (fluo-4 acetoxymethyl ester; Molecular Probes) was added to the culture. ATP (10 μm) was used to trigger intracellular calcium signal. Click here for file

Additional file 10: Video 8 Detection of intracellular Ca^2+ ^signals with confocal microscopy – APAP-treated primary hepatocytes displayed repeated calcium signal following ATP administration. Nuclear and cytosolic Ca^2+ ^were monitored in individual cells by using time-lapse confocal microscopy. Primary mouse hepatocytes were cultured on glass coverslips in a density of 3×10^5 ^cells/well in 6 wells plates and kept in a Hepes-buffered solution during experiments. Cells were incubated with APAP (20 mM) and after 6 hours 4 μM cell permeant Fluo4-AM (fluo-4 acetoxymethyl ester; Molecular Probes) was added to the culture. ATP (10 μm) was used to trigger intracellular calcium signal. Click here for file

Additional file 11: Video 9 Detection of intracellular Ca^2+ ^signals with confocal microscopy – responsiveness to exogenous ATP. Nuclear and cytosolic Ca^2+ ^were monitored in individual cells by using time-lapse confocal microscopy. Primary mouse hepatocytes were cultured on glass coverslips in a density of 3×10^5 ^cells/well in 6 wells plates and kept in a Hepes-buffered solution during experiments. Cells were incubated with APAP (20 mM) and suramin (0.1 mM) and after 6 hours 4 μM cell permeant Fluo4-AM (fluo-4 acetoxymethyl ester; Molecular Probes) was added to the culture. ATP (10 μm) was used to trigger intracellular calcium signal. Click here for file

## References

[B1] Bernal W Auzinger G Dhawan A Wendon J Acute liver failure Lancet 2010 376 190 201 10.1016/S0140-6736(10)60274-7 20638564

[B2] Rolando N Wade J Davalos M Wendon J Philpott-Howard J Williams R The systemic inflammatory response syndrome in acute liver failure Hepatology 2000 32 734 739 11003617 10.1053/jhep.2000.17687

[B3] Hinson JA Roberts DW James LP Mechanisms of acetaminophen-induced liver necrosis Handb Exp Pharmacol 2010 196 369 405 10.1007/978-3-642-00663-0_12 20020268 PMC2836803

[B4] Chen GY Nunez G Sterile inflammation: sensing and reacting to damage Nat Rev Immunol 2010 10 826 837 10.1038/nri2873 21088683 PMC3114424

[B5] Rock KL Latz E Ontiveros F Kono H The sterile inflammatory response Annu Rev Immunol 2009 28 321 342 20307211 10.1146/annurev-immunol-030409-101311 PMC4315152

[B6] Ahrens S Zelenay S Sancho D Hanc P Kjaer S Feest C Fletcher G Durkin C Postigo A Skehel M F-Actin Is an Evolutionarily Conserved Damage-Associated Molecular Pattern Recognized by DNGR-1, a Receptor for Dead Cells Immunity 2012 36 635 645 10.1016/j.immuni.2012.03.008 22483800

[B7] Bravo R Gutierrez T Paredes F Gatica D Rodriguez AE Pedrozo Z Chiong M Parra V Quest AF Rothermel BA Lavandero S Endoplasmic reticulum: ER stress regulates mitochondrial bioenergetics Int J Biochem Cell Biol 2012 44 16 20 10.1016/j.biocel.2011.10.012 22064245 PMC4118286

[B8] Grimm S The ER-mitochondria interface: the social network of cell death Biochim Biophys Acta 2012 1823 327 334 10.1016/j.bbamcr.2011.11.018 22182703

[B9] Glancy B Balaban RS Role of mitochondrial Ca2+ in the regulation of cellular energetics Biochemistry 2012 51 2959 2973 10.1021/bi2018909 22443365 PMC3332087

[B10] Martinon F Burns K Tschopp J The inflammasome: a molecular platform triggering activation of inflammatory caspases and processing of proIL-beta Mol Cell 2002 10 417 426 10.1016/S1097-2765(02)00599-3 12191486

[B11] Hoque R Sohail MA Salhanick S Malik AF Ghani A Robson SC Mehal WZ P2X7 receptor-mediated purinergic signaling promotes liver injury in acetaminophen hepatotoxicity in mice Am J Physiol Gastrointest Liver Physiol 2012 302 G1171 G1179 10.1152/ajpgi.00352.2011 22383490 PMC3362096

[B12] Corcoran GB Wong BK Neese BL Early sustained rise in total liver calcium during acetaminophen hepatotoxicity in mice Res Commun Chem Pathol Pharmacol 1987 58 291 305 3438568

[B13] Salas VM Corcoran GB Calcium-dependent DNA damage and adenosine 3',5'-cyclic monophosphate-independent glycogen phosphorylase activation in an in vitro model of acetaminophen-induced liver injury Hepatology 1997 25 1432 1438 10.1002/hep.510250621 9185764

[B14] Leite MF Thrower EC Echevarria W Koulen P Hirata K Bennett AM Ehrlich BE Nathanson MH Nuclear and cytosolic calcium are regulated independently Proc Natl Acad Sci U S A 2003 100 2975 2980 10.1073/pnas.0536590100 12606721 PMC151451

[B15] Burnstock G Purinergic signalling: past, present and future Braz J Med Biol Res 2009 42 3 8 10.1590/S0100-879X2008005000037 18853040

[B16] McDonald B Pittman K Menezes GB Hirota SA Slaba I Waterhouse CC Beck PL Muruve DA Kubes P Intravascular danger signals guide neutrophils to sites of sterile inflammation Science 2010 330 362 366 10.1126/science.1195491 20947763

[B17] Marques PE Amaral SS Pires DA Nogueira LL Soriani FM Freire Lima BH Oliveira Lopes GA Russo RC Avila TV Melgaco JG Chemokines and mitochondrial products activate neutrophils to amplify organ injury during mouse acute liver failure Hepatology 2012 56 1971 1982 10.1002/hep.25801 22532075

[B18] Milne GR Palmer TM Anti-inflammatory and immunosuppressive effects of the A2A adenosine receptor Scientific World Journal 2011 11 320 339 21298223 10.1100/tsw.2011.22 PMC5720067

[B19] Tschopp J Schroder K NLRP3 inflammasome activation: The convergence of multiple signalling pathways on ROS production? Nat Rev Immunol 2010 10 210 215 10.1038/nri2725 20168318

[B20] Iyer SS Pulskens WP Sadler JJ Butter LM Teske GJ Ulland TK Eisenbarth SC Florquin S Flavell RA Leemans JC Sutterwala FS Necrotic cells trigger a sterile inflammatory response through the Nlrp3 inflammasome Proc Natl Acad Sci U S A 2009 106 20388 20393 10.1073/pnas.0908698106 19918053 PMC2787135

[B21] Luheshi NM Giles JA Lopez-Castejon G Brough D Sphingosine regulates the NLRP3-inflammasome and IL-1beta release from macrophages Eur J Immunol 2012 42 716 725 10.1002/eji.201142079 22105559 PMC3491674

[B22] Kozlov S Gueven N Keating K Ramsay J Lavin MF ATP activates ataxia-telangiectasia mutated (ATM) in vitro. Importance of autophosphorylation J Biol Chem 2003 278 9309 9317 10.1074/jbc.M300003200 12645530

[B23] Pelegrin P Surprenant A Dynamics of macrophage polarization reveal new mechanism to inhibit IL-1beta release through pyrophosphates EMBO J 2009 28 2114 2127 10.1038/emboj.2009.163 19536133 PMC2699392

[B24] Lazarowski ER Boucher RC Harden TK Mechanisms of release of nucleotides and integration of their action as P2X- and P2Y-receptor activating molecules Mol Pharmacol 2003 64 785 795 10.1124/mol.64.4.785 14500734

[B25] Bours MJ Dagnelie PC Giuliani AL Wesselius A Di Virgilio F P2 receptors and extracellular ATP: a novel homeostatic pathway in inflammation Front Biosci (Schol Ed) 2011 3 1443 1456 21622280 10.2741/235

[B26] Murakami T Ockinger J Yu J Byles V McColl A Hofer AM Horng T Critical role for calcium mobilization in activation of the NLRP3 inflammasome Proc Natl Acad Sci U S A 2012 109 11282 11287 10.1073/pnas.1117765109 22733741 PMC3396518

[B27] Graubardt N Fahrner R Trochsler M Keogh A Breu K Furer C Stroka D Robson SC Slack E Candinas D Beldi G Promotion of liver regeneration by natural killer cells in mice is dependent on extracellular ATP phosphohydrolysis Hepatology 2012 in press 10.1002/hep.26008 22898900

[B28] Schlosser SF Burgstahler AD Nathanson MH Isolated rat hepatocytes can signal to other hepatocytes and bile duct cells by release of nucleotides Proc Natl Acad Sci U S A 1996 93 9948 9953 10.1073/pnas.93.18.9948 8790437 PMC38535

[B29] Patel SJ Milwid JM King KR Bohr S Iracheta-Velle A Li M Vitalo A Parekkadan B Jindal R Yarmush ML Gap junction inhibition prevents drug-induced liver toxicity and fulminant hepatic failure Nat Biotechnol 2012 30 179 183 10.1038/nbt.2089 22252509 PMC3609650

[B30] Ayata CK Ganal SC Hockenjos B Willim K Vieira RP Grimm M Robaye B Boeynaems JM Di Virgilio F Pellegatti P Purinergic P2Y(2) Receptors Promote Neutrophil Infiltration and Hepatocyte Death in Mice With Acute Liver Injury Gastroenterology 2012 143 1620 1629 e1624 10.1053/j.gastro.2012.08.049 22974709

[B31] Xie Y Williams CD McGill MR Lebofsky M Ramachandran A Jaeschke H Purinergic Receptor Antagonist A438079 Protects Against Acetaminophen-induced Liver Injury by Inhibiting P450 Isoenzymes not Inflammasome Activation Toxicol Sci 2012 131 325 335 22986947 10.1093/toxsci/kfs283 PMC3537131

[B32] Zampese E Pizzo P Intracellular organelles in the saga of Ca2+ homeostasis: different molecules for different purposes? Cell Mol Life Sci 2012 69 1077 1104 10.1007/s00018-011-0845-9 21968921 PMC11114864

[B33] Liu ZX Han D Gunawan B Kaplowitz N Neutrophil depletion protects against murine acetaminophen hepatotoxicity Hepatology 2006 43 1220 1230 10.1002/hep.21175 16729305

[B34] Zhang Q Raoof M Chen Y Sumi Y Sursal T Junger W Brohi K Itagaki K Hauser CJ Circulating mitochondrial DAMPs cause inflammatory responses to injury Nature 2010 464 104 107 10.1038/nature08780 20203610 PMC2843437

[B35] Amaral FA Costa VV Tavares LD Sachs D Coelho FM Fagundes CT Soriani FM Silveira TN Cunha LD Zamboni DS NLRP3 inflammasome-mediated neutrophil recruitment and hypernociception depend on leukotriene B(4) in a murine model of gout Arthritis Rheum 2012 64 474 484 10.1002/art.33355 21952942

[B36] Russo RC Garcia CC Barcelos LS Rachid MA Guabiraba R Roffe E Souza AL Sousa LP Mirolo M Doni A Phosphoinositide 3-kinase gamma plays a critical role in bleomycin-induced pulmonary inflammation and fibrosis in mice J Leukoc Biol 2011 89 269 282 10.1189/jlb.0610346 21048214

[B37] Saito C Zwingmann C Jaeschke H Novel mechanisms of protection against acetaminophen hepatotoxicity in mice by glutathione and N-acetylcysteine Hepatology 2010 51 246 254 10.1002/hep.23267 19821517 PMC2977522

[B38] Nakamura K Mizutani R Sanbe A Enosawa S Kasahara M Nakagawa A Ejiri Y Murayama N Miyamoto Y Torii T Evaluation of drug toxicity with hepatocytes cultured in a micro-space cell culture system J Biosci Bioeng 2011 111 78 84 10.1016/j.jbiosc.2010.08.008 20837398

[B39] Zuckman DM Hung JB Roy CR Pore-forming activity is not sufficient for Legionella pneumophila phagosome trafficking and intracellular growth Mol Microbiol 1999 32 990 1001 10.1046/j.1365-2958.1999.01410.x 10361301

[B40] Resende RR Adhikari A da Costa JL Lorencon E Ladeira MS Guatimosim S Kihara AH Ladeira LO Influence of spontaneous calcium events on cell-cycle progression in embryonal carcinoma and adult stem cells Biochim Biophys Acta 2010 1803 246 260 10.1016/j.bbamcr.2009.11.008 19958796

[B41] Resende RR da Costa JL Kihara AH Adhikari A Lorencon E Intracellular Ca2+ regulation during neuronal differentiation of murine embryonal carcinoma and mesenchymal stem cells Stem Cells Dev 2010 19 379 394 10.1089/scd.2008.0289 19032055

[B42] Cruz LN Guerra MT Kruglov E Mennone A Garcia CR Chen J Nathanson MH Regulation of multidrug resistance-associated protein 2 by calcium signaling in mouse liver Hepatology 2010 52 327 337 10.1002/hep.23625 20578149 PMC3025771

[B43] Hernandez E Leite MF Guerra MT Kruglov EA Bruna-Romero O Rodrigues MA Gomes DA Giordano FJ Dranoff JA Nathanson MH The spatial distribution of inositol 1,4,5-trisphosphate receptor isoforms shapes Ca2+ waves J Biol Chem 2007 282 10057 10067 10.1074/jbc.M700746200 17284437 PMC2825872

