# Supplementary figures and images for: Altered responsiveness to extracellular ATP enhances acetaminophen hepatotoxicity
Source: Cell Commun Signal. 2013 Feb 5;11:10. doi: 10.1186/1478-811X-11-10 (PMC3608937; doi:10.1186/1478-811X-11-10)

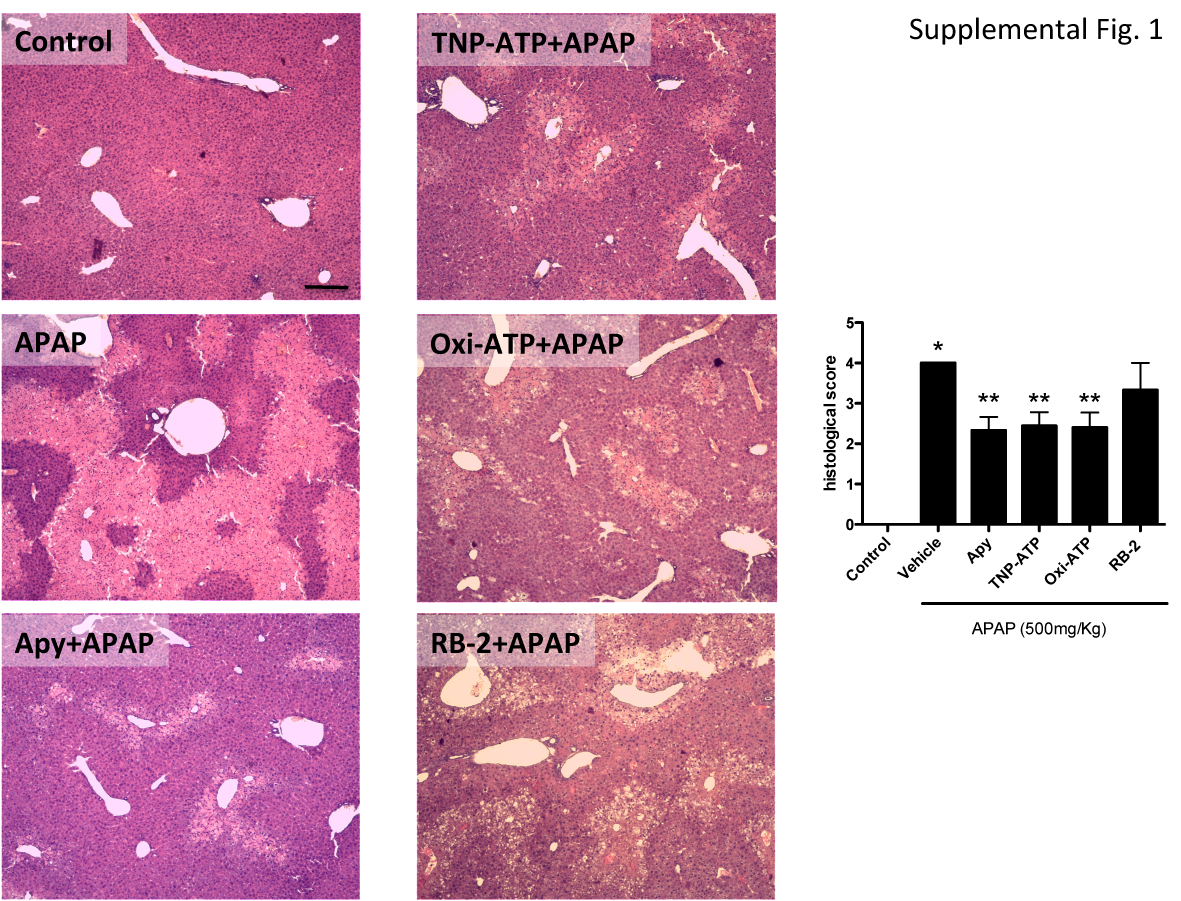

Supplement: Additional file 4: Figure S1 — H&E slides and histological score from mice treated with different P2R antagonists and challenged with APAP. Mice were treated (2 hours after APAP challenge; 500 mg/Kg; 24 h) with apyrase (25 U/mice), TNP-ATP (1 mg/Kg; i.v.), oxidized-ATP (oxi-ATP; 9 mg/Kg; i.v.) or reactive blue-2 (10-100 mg/Kg; i.p.). Histological score was assessed using 0: no lesion present; 1/2: individual necrotic cells seen at the first cell layer adjacent to the central vein, and hyaline degeneration present; 1: necrotic cells extending two or three cell layers from the central veins; 2: necrotic cells extending three to six cell layers from the central veins, but limited in peripheral distribution; 3: the same as 2, but with necrosis extending from one central vein to another; 4: more severe than 3, with extensive centrilobular necrosis throughout the section. A final score was given for each liver section. * - P < 0.05 in comparison to control (C) group and ** in comparison to vehicle treated group. Data are mean ± SEM. Scale: 100 μm. [file 1478-811X-11-10-S4.tiff]

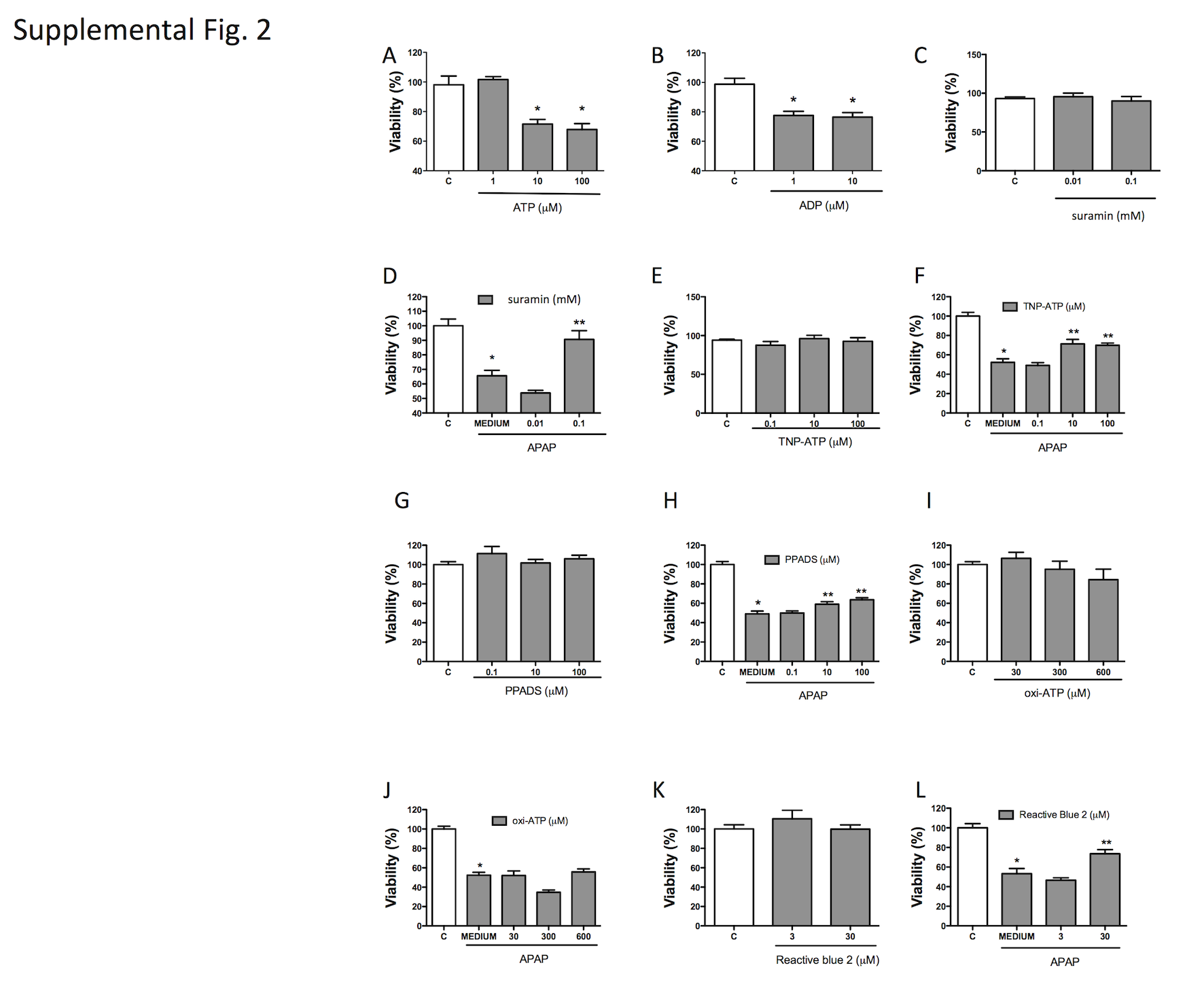

Supplement: Additional file 5: Figure S2 — Dose–response curves of ATP, ADP and different purinergic receptors antagonists. (A) Dose–response curve ATP and (B) ADP incubation upon HepG2 cells. (C-D) Dose–response curves of suramin (non-selective P2 antagonist; alone and with APAP), (E-F) TNP-ATP (P2X blocker; alone and with APAP), (G-H) PPADS (P2X blocker; alone and with APAP), (I-J) oxi-ATP (specific P2X7 blocker; alone and with APAP) or (K-L) reactive blue-2 (P2Y blocker; alone and with APAP). * - P < 0.05 in comparison to control (C) group and ** in comparison to medium treated group. [file 1478-811X-11-10-S5.tiff]
